# Supplementary material for: DMRforPairs: identifying Differentially Methylated Regions between unique samples using array based methylation profiles
Source: BMC Bioinformatics. 2014 May 15;15:141. doi: 10.1186/1471-2105-15-141 (PMC4046028; doi:10.1186/1471-2105-15-141)
Supplement: Additional file 2 — DMRforPairs output for the comparison of A431-MCF7 and NA17018-NA17105. Please start from the HTML files in each folder. Available via the BMC Bioinformatics website. [file 1471-2105-15-141-S2.zip › 1394847754114233_MOESM2_ESM/A431_MCF7/figures/31603.pdf]

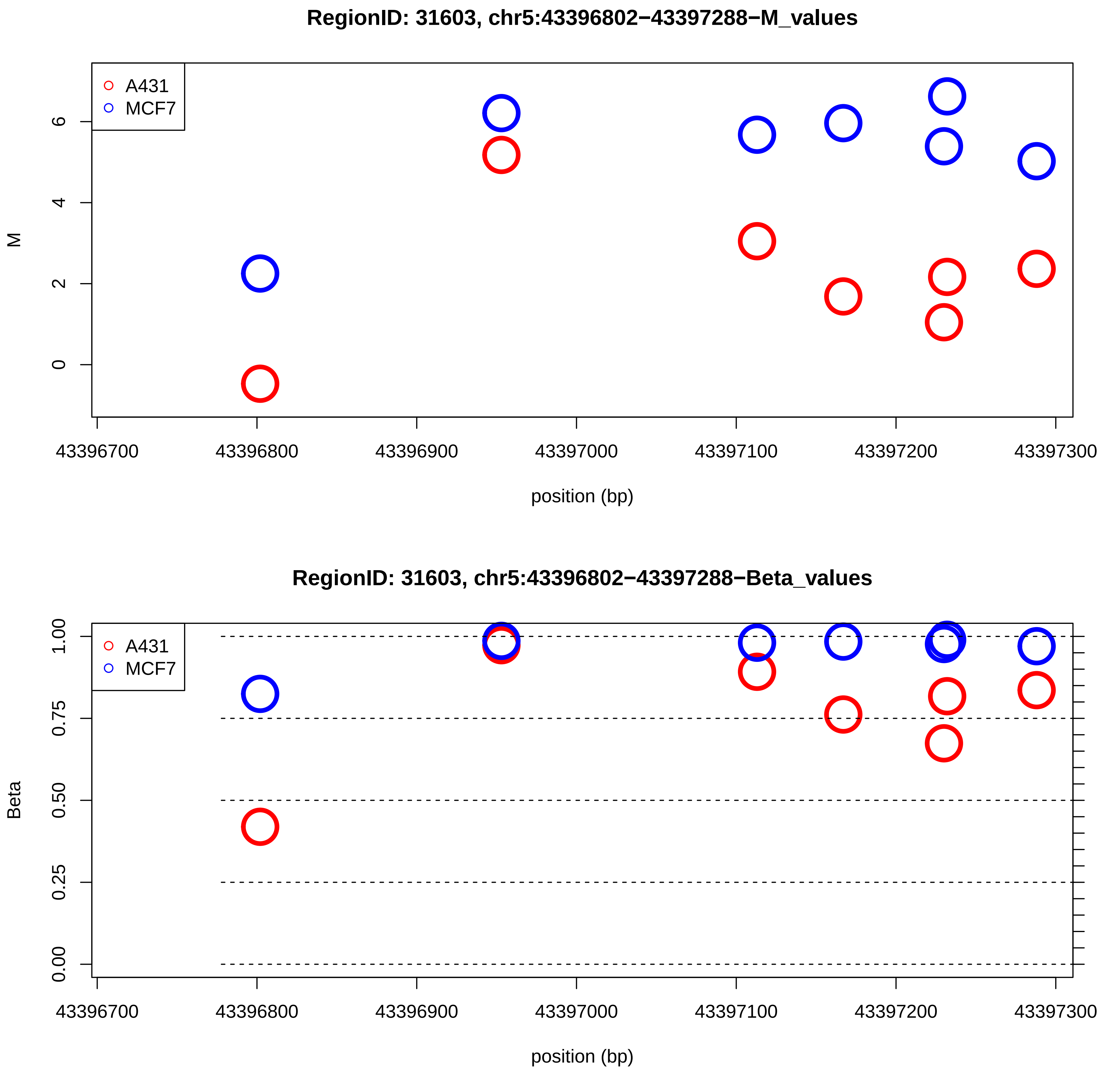

RegionID: 31603, chr5:43396802−43397288−M_values
A431
MCF7
M
0
2
4
6
43396700
43396800
43396900
43397000
43397100
43397200
43397300
position (bp)
RegionID: 31603, chr5:43396802−43397288−Beta_values
A431
MCF7
Beta
0.00
0.25
0.50
0.75
1.00
43396700
43396800
43396900
43397000
43397100
43397200
43397300
position (bp)

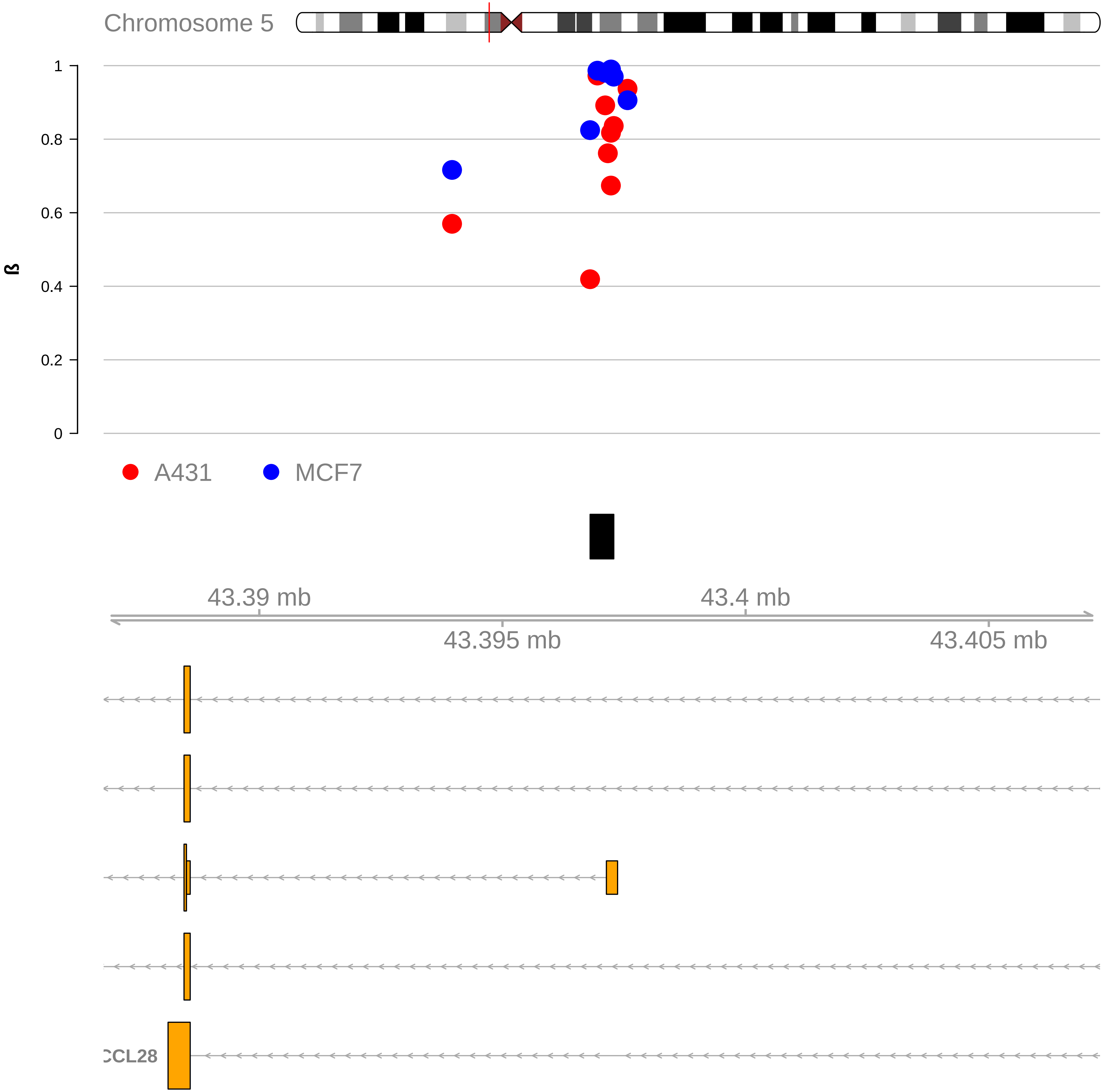

Chromosome 5
1
0.8
0.6
0.4
0.2
0
ß
A431
MCF7
43.39 mb
43.4 mb
43.395 mb
43.405 mb
CCL28
